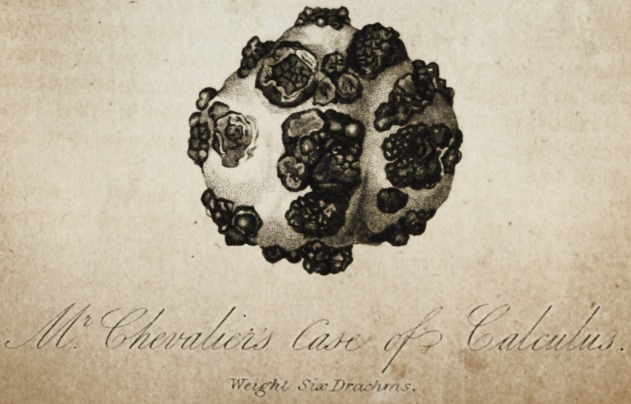# Case of Urinary Calculus, Removed by Dilatation of the Female Urethra

**Published:** 1826-09

**Authors:** 


Mr. Chevalier's Case of Calculus.

### URINARY CALCULUS.

*Case of Urinary Calculus, removed by Dilatation of the Female Urethra, in a Patient treated at the* Westminster General Dispensary. Communicated by T. Wm. Chevalier, Esq.

[with an engraving.]

Ann F—, ætatis sixteen, a girl of small stature and feeble habit, with quick and irritable pulse, and every usual mark of a delicate constitution, applied to me, as Mr. Copland Hutchison's *locum tenens* at the Westminster General Dispensary, in January 1825. According to her mother's account, she had complained of occasional lancinating pains about the bladder, especially when she made water, for eleven years. She told me herself that this symptom, together with those of pain in the loins and reins, and the frequent appearance of blood in her water, had become so severe during the last two years as to render her life miserable,—more especially of late, from her having become liable to incontinence of urine. She had exhibited as yet no symptom of puberty; unless, indeed, a hard tumor, of irregular shape, and as large as a small walnut, in the place of the right mammary gland. She had been sounded at the Middlesex Hospital, about eighteen months since, and a stone was found in her bladder; but she then refused to submit to any operation. Her bowels of late had become obstinately costive.

On the 30th of January, I examined her a second time, in the presence of Mr. Harding, when the calculus represented in the plate was distinctly to be felt, both with the female sound and by the finger introduced per vaginam.

At two o'clock in the afternoon, her bowels having been previously opened, the patient was placed upon her back on the bed; and, the smallest dilator urethræ being found considerably too large to pass, I introduced into the bladder a phimosis dilator, and with this instrument I felt the calculus. The first attempt to open the blades by the action of the screw gave considerable pain, which continued to increase until their widest divergency, to the extent of three-fourths of an inch, was accomplished; viz. in the space of half an hour, at intervals of from two to three, or four, minutes. Some blood had oozed, and the sufferings of the patient were become exceedingly severe. Considerable force upon the screw was required after every pause, to effect further dilatation; and the sides of the urethra appeared as tense as although ready to tear.

The dilator urethræ was now introduced with the blades closed, in which position they composed a cylinder of half an inch in diameter. This instrument could not yet be passed into the bladder; for the blades of the former, as well as these also, were so insufficiently firm, that their extremities could not be separated within the neck of the bladder to nearly the same extent as their bases. The dilatation was now continued, therefore, *without* the neck of the bladder, to the extent of an inch; and afterwards this instrument was introduced, with the blades closed, into the bladder, to feel the stone; and the blades separated to their widest extent,—viz. to an inch and three-quarters. The pain, since the first introduction of this latter instrument, was as severe as can well be conceived, being as violent as any bodily suffering I ever saw endured under any circumstances, and altogether without remission. I now introduced my fore-finger into the bladder, not without difficulty, for the urethra contracted forcibly upon removing the distention by the dilator. I was happy to find that, notwithstanding the oozing of about two drachms of blood, the surface of the urethra was merely abraded; for the only assurance I had had hitherto of the urethra remaining untorn, was from constant attention to every sensation conveyed along the instrument, and from the continuing stress upon it. The instrument last mentioned being the largest I had with me, (for I was not aware of the insufficiency of the strength of its blades, especially as it had been used before in the extraction of as large a stone,) I now dilated the parts by expanding the blades, with more and more rapidity, in different directions; and by this means the urethra was enlarged to a considerably further extent than by their first expansion.

Although the calculus could now be felt with ease, and turned in any direction, the canal was found to be still too narrow (especially at the neck of the bladder, where the blades of the dilator yielded so inconveniently,) to admit of its exit; and I therefore continued to increase the distention by means of my fore-finger and the closed forceps, until at length, at half-past five o'clock, it became practicable to bring the stone into the urethra. After this was accomplished, however, it was not to be withdrawn without great difficulty; so much so, that the interior part of the lining membrane of the urethra was brought down into view, and in a slight degree everted, and I was obliged, with my nail, to turn off its folds from several of the noduli represented in the plate upon the body of the calculus.

The patient, being extremely exhausted, was allowed to take a little wine and water. The urethra was abraded, not by the stone, but by the instrument, over which it had been so extremely tense, during the distention.

At seven o'clock, I prescribed Tr. Opii et Vini Antimonii Tartarizati āā gtt. xx.; and, in the course of the evening, my patient took half an ounce of Sulphate of Magnesia. The parts were frequently washed with warm milk and water, and anointed with lard, both internally and externally.

In the forenoon of the next day, the bowels had not been relieved; her tongue was much furred; the pulse 130, and hard; the skin hot; the abdomen tender as far as the umbilicus; the urethra very painful, from the constant flow of urine. She had drank freely of linseed-tea.

She now took four grains of Calomel, and, at intervals of four hours, two doses of Sulphate of Magnesia; and twelve leeches were applied to the abdomen, to be succeeded by poppy fomentations.

In the evening, the tenderness was increased, as well as the heat of skin and irritation in the urethra; but the pulse was softer, and more varying in frequency; and, as the leech-bites were still bleeding, and the aperients just beginning to operate, I left her for the night, prescribing only a perseverance in the use of fomentations.

On the morning of the next day, (February 1st,) the tenderness of the abdomen was completely removed; the leech-bites having continued to bleed gently for nine hours from the time they were applied. The bowels had been freely opened; the pulse was soft, varying from 120 to 130; the skin hot, but moist.

She was ordered to take four grains of Antimonial Powder every twelfth hour.—In the evening, the Epsom Salts were repeated.

Feb. 2d.—Temper more irritable; tongue white; pulse 120, and hard; abdomen not at all tender.

I allowed her to eat some fish; but ordered the Antimonial to be repeated every sixth hour.

—A very superficial slough appeared to-day at the orifice of the urethra: it came away on the morrow, leaving the canal, to all appearance, perfectly healthy.

3d.—From this date she continued to improve under the use of Antimony and Extractum Hyoscyami; with attention to her bowels, which were still obstinately costive.

On the 12th, she made about three ounces of urine by a voluntary effort; and, for the first time in her life, the catamenia appeared.

She made from a quarter to half a pint of urine on several occasions by voluntary efforts, on the 13th, the orifice of the urethra having nearly regained its ordinary dimensions; and on the 17th, she was to all appearance perfectly cured.

On the two following days, however, she suffered from occasional incontinence of urine; and, on the 20th, retention of urine occurred, and continued for twelve hours, until at length a small slough came away, and, to my great disappointment, there ensued incontinence of urine almost complete.

Under the use of nourishing diet, of wine, and the mineral tonics, she gradually improved; at first, by recovering the power of retaining her urine through the greater part of the night, and then the ability to void it voluntarily sometimes during the day: it took upwards of three months, however, for her complete recovery from the incontinence, and a still longer period elapsed before she was able to retain above half a pint of fluid in the bladder, during the day; so that she became a strong healthy girl, and was fully formed, before the local effects of the disease and of the operation had entirely ceased. At length, however, she recovered completely, and has enjoyed the most perfect health for many months past; the tumor in the right breast having proved to be nothing more than an irregular development of the mammary gland.

The annexed drawing is made by measurement, and therefore accurately exhibits the size of the calculus, as well as its extremely irritating and irregular surface: its largest circumference is three inches and nine-tenths; its smallest circumference is three inches and nine-twentieths. On its section there appears a nucleus composed of lithate of ammonia and oxalate of lime, of from three to four-tenths diameter. This is surrounded by a mulberry calculus, projecting in the noduli seen in the plate, and of unusual purity, as I am informed by Dr. Prout, who has analysed the stone. The surface represented white in the drawing is composed of the triple phosphate, and its greatest depth is about one-seventh of an inch.*

Before I conclude this communication, I may be allowed to offer a few remarks upon the account which I have given of the foregoing case. In the first place are to be observed the unfavourable shape and large size of the calculus; the retarded, or rather the obviated, maturity of the patient's form, which was so incomplete that the vagina itself was not half the size or capacity to which it was necessary to dilate the urethra. Her constitution, also, being rendered irritable, not only by the disease, but also by the prevention of the usual phenomena of her age; while her digestive organs had become habitually inactive, and her mind was in a state of complete despondency, in consequence of the afflicting circumstance of incontinence of urine.

Much consideration was given to her case before the operation was performed; and it was from the general, if not universal, truth, that severer pain for a shorter time is more endurable, and of less injurious consequence, than somewhat

* See Medico-Chir. Transactions, vol. x. p. 389.

less severe suffering continued for a longer time, that I was induced to resolve upon as speedy an operation as could be accomplished without mechanical injury. In this resolution I was moreover confirmed, by an assurance that one of the first surgeons in this metropolis had expressed the above opinion, with intentional and direct reference to this operation; and also by the fact, that that gentleman's cases, as well as Mr. A. C. Hutchison's cases of this operation, had been performed in a very short time with perfect success. The experience of the profession on this point being so limited, it is not surprising that my instruments were less adapted to the individual case than they may be made; or that some perplexing circumstances occurred in a case so completely novel.

My object in publishing it is to prove that there is danger in dilating the female urethra too rapidly; and even greater danger than in inflicting the severest pain for many hours. For inflammation of the peritoneum from mechanical injury to a neighbouring part, is a manageable disease, in comparison to sloughing produced by such injury in an enfeebled and irritable constitution: and, indeed, I have heard of the case of a patient who died of extensive and incontrollable sloughing, after this operation performed in two hours.

The benefit which my patient has derived, although perhaps more slowly obtained than it might be (after the experience of her case) in another, is yet most complete; and whereas her life was a burden to her, and also imminently endangered, she has now the full enjoyment of health.

I am sure, however, that no patient would submit to this operation, if previously aware of the suffering which was to be endured; unless, indeed, upon the alternative of certainly dying of the disease, after protracted torture, or of submitting to the evil of incontinence of urine, after the operation with the knife; and, judging from this single case, I am willing to express my opinion, that we are not justified in inflicting so much suffering (instead of proposing the operation by the bistoire caché), excepting by the fact that the patient must endure incontinence of urine, in all probability, for her whole life, if operated on by any other means than the dilator.

The evils of a disability to retain the urine are to a female so great, that even the chance of them may make it well worth her while to undergo any pain as an alternative: yet I would respectfully suggest to the profession, whether it may not be worthy of their consideration to perform the operation of lithotomy above the pubes, in preference to this operation, in the cases of females? The operation of lithotomy above the pubes has succeeded over and over again; and, although it has failed often, may there not have been manifest and avoidable causes of its failure?

With proper management, it must be a safer operation in the female than in the male; and I know nothing to show that it might not be perfectly safe, and perfectly successful—while it is only of a few minutes' duration.

20, *South Audley-street; July* 18*th*, 1826.

---

## Figures and Tables

**Figure f1:**